# Effects of Temperature on the Developmental and Reproductive Biology of North American Bean Thrips, *Caliothrips fasciatus* (Pergande) (Thysanoptera: Thripidae: Panchaetothripinae)

**DOI:** 10.3390/insects14070641

**Published:** 2023-07-15

**Authors:** Mark S. Hoddle, Ivan Milosavljević, Ruth Amrich

**Affiliations:** 1 Department of Entomology, University of California Riverside, Riverside, CA 92521, USA; mark.hoddle@ucr.edu (M.S.H.); ruth.amrich@ucr.edu (R.A.); 2 Citrus Research Board, Visalia, CA 93279, USA

**Keywords:** accumulative cold stress, citrus, fruit exports, propagule pressure, quarantine pest

## Abstract

**Simple Summary:**

North American bean thrips, *Caliothrips fasciatus*, native to California (U.S.), is a regular contaminant of navel oranges exported from California. Despite more than 100 years of accidental shipments overseas, this polyphagous thrips has not established outside of its native range. Understanding why this thrips has not successfully invaded other parts of the world may be explained, in part, by the effects of temperature on fitness. This study investigated the effects of nine fluctuating temperatures that averaged 8, 10, 15, 20, 25, 30, 32, 35, and 37 °C over a 24 h period on the developmental and reproductive biology of *C. fasciatus* to better understand the effects of temperature on these critical life history characters and how temperature may influence the invasion potential of this thrips.

**Abstract:**

North American bean thrips, *Caliothrips fasciatus*, native to California U.S., has been detected inside the navels of navel oranges exported from California for more than 120 years. Despite this long history of accidental movement into new areas, this thrips has failed to establish populations outside of its native range. The cold accumulation hypothesis postulates that increasing levels of cold stress experienced by thrips overwintering inside navels is compounded when harvested fruit is shipped under cold storage conditions. Consequently, the fitness of surviving thrips is compromised, which greatly diminishes invasion potential. At the time this study was conducted, the effects of temperature on *C. fasciatus* fitness were unknown. To address this shortcoming, the effects of nine fluctuating temperatures that averaged 8, 10, 15, 20, 25, 30, 32, 35, and 37 °C over a 24 h period on the developmental and reproductive biology of *C. fasciatus* were evaluated. One linear and five nonlinear regression functions were fit to egg-to-adult development rate data for parent and offspring thrips to characterize thermal performance curves. Estimates of minimum, optimal, and maximum temperature thresholds for development were in the ranges of −4.37–6.52 °C (i.e., *T*_min_), 31.19–32.52 °C (i.e., *T*_opt_), and 35.07–37.98 °C (i.e., *T*_max_), respectively. Degree day accumulation to complete development, estimated from linear regression, ranged 370.37–384.61. Average development times for eggs, first and second instar larvae, propupae, pupae, and adult longevity, and mean lifetime fecundity of females were significantly affected by temperature. These biological responses to temperature may provide insight into how this abiotic variable affects the invasion potential of *C. fasciatus*.

## 1. Introduction

North American bean thrips, *Caliothrips fasciatus* (Pergande, 1895) (Thysanoptera: Thripidae), is native to California (U.S.) and has a natural range that extends north from California into western Canada, south into México, and east into Idaho and Florida (both U.S. states) [1]. Confirmation of putative breeding populations of *C. fasciatus* outside of this native range in Puerto Rico, Hawai’i [U.S.], Argentina, Brazil, and China are lacking [2,3]. In California, *C. fasciatus* is widely distributed and breeds on various native and introduced weed species including sow thistle (*Sonchus oleraceus* L.), prickly lettuce (*Latuca scariola* L.), cheese weed (*Malva parviflora* L.), fennel (*Foeniculum vulgare* Miller), and California poppy (*Eschscholzia californica* Cham.) [4,5,6,7].

Historically, *C. fasciatus* was a significant agricultural pest in California, infesting alfalfa (*Medicago sativa* L.), beans (*Phaseolus vulgaris* L.), cantaloupes (*Cucumis melo* L.), cotton (*Gossypium hirsutum* L.), lettuce (*Lactuca sativa* L.), pear (*Pyrus* spp.), pea (*Pisum sativum* L.), and walnut (*Juglans regia* L.) [4,5,6]. Curiously, pestiferousness has waned significantly, possibly due to development of integrated pest management programs, use of insecticides with greater efficacy, or development of resistance cultivars [8]. Currently, the major agricultural issue associated with *C. fasciatus* in California is trade related. In California, adult *C. fasciatus* overwinter inside the “navels” of navel oranges from early November to late March [3]. Infested fruit are harvested over this time period and exported. Detections of *C. fasciatus* concealed within fruit exported from California trigger quarantine procedures when intercepted by importing countries (e.g., Australia and New Zealand). In some years ~6% of imported shipments may be contaminated with live thrips [2,7]. *Caliothrips fasciatus* is amongst the 10 most frequently border-intercepted Thysanoptera for Australia (over the period 2003–2016) and New Zealand (2000–2017) (Rebecca Turner pers. comm. 2023). To mitigate quarantine issues, research on *C. fasciatus* has focused, in part, on the development of sampling, monitoring, and management programs in citrus [2,9,10] and fumigation strategies for harvested fruit [11,12].

Interestingly, exports of fresh citrus fruit contaminated with overwintering *C. fasciatus* have been occurring for >120 years. *Caliothrips fasciatus* was detected in navel oranges exported from California to Nebraska, Illinois, and Hawai’i (all U.S. states) as early as 1899, 1907, and 1929, respectively [3]. Therefore, it is likely that *C. fasciatus* has been unintentionally and widely distributed in navel oranges exported from California, the largest producer of this fruit in the U.S. [13], for a long period of time. Amongst the top 15 importing markets for fresh U.S. grown oranges in 2021 (South Korea was ranked number 1) are Australia (ranked 7) and New Zealand (ranked 15) [14]. The navel orange marketing season in California is 1 October–15 June [13], with the bulk of exports to Australia and New Zealand, for example, occurring over the period November–March, which spans meteorological fall–winter in California. This export period coincides mainly with meteorological spring–summer in Australia and New Zealand, a time of year when temperatures would be favorable for *C. fasciatus* establishment, and host plants in a suitable phenological state for infestation would be abundant [7].

Propagule pressure, a function of frequency of introductions and numbers of individuals introduced each time [15,16,17], is a reliable predictor of establishment likelihood [16,17]. For example, low propagule pressure (<10 individuals) results in low establishment rates due to Allee effects (e.g., inability to locate mates) or environmental stochasticity (e.g., inclement weather events) [16,17]. In contrast, propagule pressure, in the range of >10–100 individuals, is associated with increased invasion success, due to greater likelihood of establishment by species introduced into new areas [16,17]. Importantly, thrips (i.e., Thysanoptera) are one of the most commonly intercepted insect orders found during border inspections [18]. Assuming that *C. fasciatus* propagule pressure has been relatively consistent, but fluctuating in intensity over time due to variations in infestation dynamics [16], a question of interest is: “Why has *C. fasciatus*, a polyphagous thrips, failed to establish outside of its native range, despite, presumably, a long history of frequent accidental introductions via importation of infested navel oranges into new areas with favorable receiving environments?” The accumulative cold stress hypothesis, in part, may provide a potential explanation for this outcome [2,7].

The accumulative cold stress hypothesis suggests that *C. fasciatus* adults suffer adverse effects from accumulating cold stress that results from overwintering inside fruit in a field prior to harvest. Cold stress accumulation increases when harvested fruit is subjected to refrigerated shipping (i.e., ~3 °C for 15–16 days in transit [7]). Collectively, accumulative cold stress from low temperature exposure in a field prior to fruit harvest, which is then followed by refrigerated shipping, may impact the fitness (e.g., longevity, fecundity, and offspring sex ratio) of surviving thrips. Consequently, live-but-cold-stressed *C. fasciatus* that make it to Australia and New Zealand, for example, may be too debilitated to establish viable populations [2,7].

Almost nothing is known about the effects of exposure that different temperature regimens have on *C. fasciatus* developmental and reproductive biology. The lack of these types of fundamental temperature-driven data is a significant impediment to investigating the effects of temperature on the fitness of *C. fasciatus* and how varying temperature exposures may affect the invasion potential of this thrips. As a first step in investigating the effects of temperature on *C. fasciatus*, work reported here was undertaken to determine the minimum, optimal, and maximum temperatures, and degree-day requirements for *C. fasciatus* to complete development and how temperature affects basic population demographic parameters such as preimaginal developmental times, adult female reproductive outputs, offspring sex ratios, and adult longevities.

## 2. Materials and Methods

### 2.1. Establishment of Thrips Colonies for Experiments

Approximately 97% of navel-orange-producing acreage in California is planted in the Central Valley, with Tulare, Kern, and Fresno Counties having 59, 22, and 18%, respectively, of the fruit-bearing acreage in the Central Valley [19]. Consequently, the major region of origin for the export of navel orange fruit contaminated with *C. fasciatus* is the Central Valley. Therefore, to ensure thrips used for initiating colonies from which material for experiments would be sourced had phenotypes representing those likely to be found contaminating citrus exported from the Central Valley, adult *C. fasciatus* were collected from Visalia and Porterville, both of which are in Tulare County, the largest county growing and exporting navel oranges. To start thrips colonies for temperature experiments, adult *C. fasciatus* were collected from foliage of asparagus, *Asparagus officinalis* L., and California poppy, *E. californica*, two very good host plants for this pest [4,5,6], which were growing in the vicinity of Visalia and Porterville, Tulare County.

To collect thrips to initiate colonies, adult *C. fasciatus* were dislodged from infested plants by gently beating foliage over a white plastic tray. Adult bean thrips were aspirated from the tray and vials of aspirated thrips were emptied into ventilated plastic boxes (25 cm × 25 cm × 40 cm) covered with 105 µm ventilation mesh that contained potted lima bean plants (*Phaseolus lunatus* L.), cultivar Fordhook 242 (Gurneys Seed and Nursery Company, Lawrenceburg, IN, USA). Colonies of *C. fasciatus* were subsequently maintained on this host plant at the University of California Riverside Insectary and Quarantine Facility inside ventilated BugDorm-2120 Insect Rearing Tents (60 cm × 60 cm × 60 cm, 160 µm ventilation mesh (Mega View Science, Taiwan)), at 26.6 °C ± 1.03 °C and 50% RH under long days (L:D 14:10 h). Plants were watered every third day and fertilized with MiracleGro^®^ (The Scotts Company LLC) at the recommended label rate. At 14-day intervals, new plants were introduced into cages, and existing plants were allowed to die, thereby forcing thrips onto new host plants. Adult thrips from this colony were collected and used to initiate the experiments detailed below.

### 2.2. Preimaginal Development, Adult Longevity, and Female Fecundity across Nine Fluctuating Temperature Regimens

To obtain thrips eggs for fluctuating temperature studies (see below), approximately 20–30 adult male–female pairs of *C. fasciatus* were removed from colony cages, and thrips pairs were placed onto the undersides (i.e., abaxial side) of clean excised Lima bean leaves, cultivar Fordhook 242, which were enclosed in labeled Munger cells [20,21]. Adult pairs of thrips were then immediately placed into temperature cabinets set for each experimental temperature regimen (see below for details on fluctuating temperature regimens). Male–female pairs were moved to new leaves every 24 h, and leaves exposed to mating pairs of thrips were removed from Munger cells, placed adaxial side (i.e., top side of leaf) down on water-saturated foam pads, and maintained at the same temperature regimen, under which oviposition had occurred. Leaves were checked daily, and the time to emergence in days for first instar thrips larvae was recorded for each experimental temperature. A minimum of 10 and a maximum of 30 first instar larvae were moved from natal leaves using a fine camel hair brush and placed individually onto clean excised Lima bean leaves, cultivar Fordhook 242, which were enclosed in labeled Munger cells. Preimaginal life stages were observed daily, and time of development in days for first and second instar larvae, propupae, and pupae were recorded for each individual thrips reared at each experimental temperature. Bailey [4] provided excellent illustrations of preimaginal and adult *C. fasciatus* life stages, and these were used to identify transitions between life stages. Feeding thrips life stages (i.e., first and second instars, and adults) were moved to fresh Lima bean leaves as this food source deteriorated. Time between leaf changes varied and was temperature dependent.

Development time of preimaginal thrips life stages (i.e., eggs, larvae, propupae, and pupae) and longevity of resultant adult male and female *C. fasciatus* in days (referred to as G1 (see below)) and fecundity of mated G1 adult female and male thrips were quantified across nine experimental temperatures that fluctuated over the course of a 24 h period (see below). To determine fecundity under fluctuating temperature regimens, mated G1 female thrips and their male partners were moved every 24 h to fresh Lima bean leaves enclosed in labeled Munger cells until death. Males that died before their female partners were replaced with similarly reared males. A minimum of 20 pairs of adult male–female pairs of thrips were used for initiating experimental cohorts for each experimental temperature. Leaves exposed to individual ovipositing G1 females, and their male partners were labeled, maintained adaxial side down on saturated foam pads, and examined every 24 h for emergence of thrips larvae. The number of larvae (referred to as G2 (see below)) emerging from leaves over time were recorded and used as an estimate of fecundity for each female by temperature regimen. G2 larvae were individually isolated in Munger cells and reared to adulthood under their natal temperature regimen to determine development times and sex ratio of progeny produced by G1 females. G2 larvae from each experimental temperature regimen that reached adulthood were set up, and a minimum of 10 male–female pairs were maintained under their experimental natal temperature conditions. Progeny production from G2 male–female pairs were used to estimate average lifetime fecundity, the mean number of males and females produced, and average egg-to-adult development times across nine fluctuating temperature regimens (see below for details). G2 thrips used for experiments were set up and maintained in a manner identical to that described above for G1 thrips.

Fluctuating temperature profiles used in experiments were representative of the Central Valley where *C. fasciatus* were collected for use in experiments and from where the majority of navel oranges and mandarins likely to be contaminated with adult thrips were exported from. To produce nine different fluctuating temperature regimes, climate-controlled cabinets (plant growth chamber model PG034; Darwin Chambers, St. Louis, MO, USA) were programmed with ramping temperature increments oscillating over a 24 h cycle so that mean daily temperatures of 8, 10, 15, 20, 25, 30, 32, 35, and 37 °C were obtained (Table 1).

Incremental steps were based on the hourly temperature profiles of days with target average temperatures from five years (2014–2018 inclusive) of field-recorded data downloaded from CIMIS weather station 39 (Latitude: 36.597444; Longitude −119.50404), located in Parlier Fresno County [22,23,24] for details on hourly temperature data calculations to determine mean daily temperature over a 24 h period). Environmental chambers were maintained at 14:10 L:D at a light intensity of 100 μE m^−2^ s^−1^, and 50% RH across all temperature profiles. Target conditions were verified by HOBO Pro V2 Temperature/RH loggers programmed to record at 15 min intervals (Onset Computer Corp., Bourne, MA, USA).

### 2.3. Statistical Analyses of Preimaginal Developmental, Female Fecundity, and Offspring Sex Ratio Data across Fluctuating Temperature Regimens

All statistical results were generated using SAS [25]. Generalized linear mixed models (GLMMs) were employed to examine the relationship between temperature and development times of G1 *C. fasciatus* offspring. The PROC GLIMMIX procedure in SAS [25] was used for the analysis. The fixed effects included in the models were the fluctuating temperature profile (ranging from 8 to 35 °C, with no development observed at 37 °C (see Results section)), sex (male or female), and their interactions. Separate models were created for each developmental stage, including *C. fasciatus* eggs, larvae, propupae, pupae, combined eggs-to-adults, and adult longevity. To account for the potential lack of statistical independence, the nesting structure incorporated G1 individuals within parent identity and temperature profile. Poisson distributions were employed for all models, based on the variances of the response variables.

Similarly, GLMMs and the PROC GLIMMIX procedure in SAS [25] were used to investigate the impact of varying temperatures on the lifetime fecundity of G1 female *C. fasciatus* and the sex ratios of adult G2 progeny. The fluctuating temperature profile, excluding 37 °C (no development occurred at this temperature (see below)), was considered as a fixed effect. The G2 *C. fasciatus* egg count, estimated from numbers of emerged larvae, followed a negative binomial distribution, whereas the G2 female-to-male sex ratio was modeled using a binomial distribution, based on the variances of the response variables. Additionally, the models included G1 female longevity and its interaction with temperature as covariates to account for their potential influence on the outcomes. To address the possible lack of statistical independence within G2 progeny from individual G1 female/male parent pairs, a nested structure was implemented. This involved considering the identity of the leaves exposed to each ovipositing G1 female and her male partner within the parent identity and temperature profile. By incorporating shared characteristics and influences within the same parent and temperature conditions, this nested approach ensured analyses were accurate.

Similar to the analysis of G1 development and adult longevity times, a GLMM was utilized to investigate the correlation between temperature and egg-to-adult development times of G2 *C. fasciatus* offspring. The PROC GLIMMIX procedure in SAS [25] was employed for this analysis. The fixed effects considered in the model were the fluctuating temperature profiles (ranging from 8 to 35 °C, with no development observed at 37 °C as stated in the Results section), sexes, and their interactions. To address potential lack of statistical independence, a nested structure was implemented for G2 individuals within G1 parent identity and temperature profile. The model utilized a Poisson distribution based on the variance of the response variable.

In all GLMMs, temperature was treated as a categorical rather than continuous variable as it exhibited a highly nonlinear relationship with each variable considered for analysis [26,27]. Pairwise comparisons for significant main effects were made using least-squared means option (SAS GLIMMIX procedure and LSMEANS statement). The Tukey–Kramer method was applied to adjust the pairwise comparisons for multiple comparisons, and a significance level of 0.05 was used for all comparisons.

Notably, no significant differences in egg-to-adult development times were observed between G1 females and G1 males, or G2 females and G2 males (see Results section; Table 2, Table 3, Table 4 and Table 5), and values were pooled by generation (i.e., G1 (pooled G1 male and G1 female data) and G2 (pooled G1 male and G1 female data)) for subsequent model fitting.

### 2.4. Fitting of Models to Temperature-Driven Caliothrips fasciatus Development Data

One linear (i.e., Ordinary Linear [28]) and seven nonlinear regression functions (i.e., Beta [29,30,31], Brière-2 [32], Lactin-2 [33,34], Lobry-Rosso-Flandrois [35,36], Performance-2 [37,38], Ratkowsky [39], and Weibull [40]) were fit to datasets to test for a relationship between fluctuating temperature profiles (see [23,24]). Models were fit only to data spanning 8 to 35 °C, as no *C. fasciatus* larvae emerged from eggs oviposited at 37 °C, and this temperature profile was omitted from regression analyses (see Table 6 for model equations and parameter definitions) and egg-to-adult development rates (1/d), which were the reciprocals of mean egg-to-adult development times (i.e., days^−1^) of G1 and G2 *C. fasciatus* (i.e., pooled male and female data). Of the seven nonlinear models evaluated, the Beta and Weibull models failed to fit development rate data for *C. fasciatus*, regardless of thrips generation, and were removed from further analyses.

Linear regression [28] and a PROC REG procedure in SAS [25] were used to calculate degree days required for egg-to-adult developmental completions of G1 and G2 *C. fasciatus* (i.e., *K*; pooled male and female data) under fluctuating temperatures and the theoretical lower development thresholds (i.e., *T*_min_). The reciprocal of the line slope quantified parameter *K*, and *T*_min_ was calculated by solving for *y* = 0 [28]. Linear model goodness-of-fit was assessed with adjusted *R*-squared (*R*^2^_adj_), where *R*^2^_adj_ > 0.9 indicates a good fit to the data (Table 4; [28]). Cook’s *D* metric was used to identify and remove influential outliers from analyses (i.e., development rate at 35 °C for both G1 and G2 thrips), where observations above a specified threshold value of *D* > 4/number of observations were classified as influential [41].

Egg-to-adult development rate data of G1 and G2 *C. fasciatus* and PROC NLIN procedure in SAS [25] were used to parameterize nonlinear models [42]. All five nonlinear models tested have four parameters and thus the same degrees of freedom [df] [43,44]. Therefore, nonlinear model goodness-of-fit was assessed with residual sum of squares (i.e., *RSS*):

$$RSS=\sum_{i=1}^{n}{\left(y_{i}-{\hat{y}}_{i}\right)}^{2}$$

where *n* represents the number of observations, and *y_i_* and *ŷ_i_* denote the observed and expected development rates at the *i*-th temperature, respectively. Nonlinear models with smallest *RSS* values indicated a better fit to the data [23,24,31,43,45]. *R*^2^_adj_ was not used to assess goodness of fit for nonlinear models, as *R*^2^_adj_ inaccurately describes the validity of a nonlinear fit to data [46].

All temperature-based development models were fitted with development rate as the response variable as data satisfied homogeneity assumptions [47]. No additional transformations of the Brière-2, Lactin-2, LRF, and Performance-2 expressions were necessary, and the least squares estimation was applied to each model in its untransformed form [47]. In the square-root (i.e., Ratkowsky) model, however, both sides of the equation were squared, so the left-hand side of the equation was the development rate rather than the square root of the rate [42]. All model outputs were graphed in SigmaPlot [48].

## 3. Results

### 3.1. Effects of Temperature on G1 Thrips Development Times

All G1 *C. fasciatus* completed development under experimental temperature profiles that ranged from 8 to 35 °C (Table 2, Table 3, Table 4 and Table 5). Mean development times of G1 *C. fasciatus* eggs, larvae, propupae, pupae, and combined egg-to-adult differed with temperature (temperature effects: *p* < 0.02 for all response variables; Table 2 and Table 3), but not with sex (sex effects: *p* > 0.36 for all response variables; Table 2), and their interactions were insignificant (sex × temperature interaction effects: *p* > 0.85 for all response variables; Table 2). Development times of G1 *C. fasciatus* eggs, larvae, propupae, pupae, and combined egg-to-adult decreased with increasing temperature, irrespective of thrips sex (Table 3).

### 3.2. Effects of Temperature on G1 Thrips Longevity Times, G1 Female Fecundity Rates, and G2 Offspring Sex Ratios

Mean longevity times of G1 *C. fasciatus* males and females differed with temperature (temperature effect; Table 2 and Table 3), but not with sex (i.e., sex effect; Table 2) and their interaction was insignificant (i.e., sex × temperature effect; Table 2). Longevity for *C. fasciatus* males and females was shortest at 8, 32 and 35 °C (Table 3). The greatest longevity for adult *C. fasciatus* males and females was observed at 10 and 15 °C (Table 3). Mean lifetime fecundity rates for G1 *C. fasciatus* females differed with temperature (F = 22.45; df1 = 7, df2 = 1160; *p* < 0.0001), with the highest numbers of G2 *C. fasciatus* eggs being produced at 20 and 25 °C (Table 4). However, these fecundity rates did not show any significant variation with G1 female longevity (F = 1.28; df1 = 1, df2 = 1160; *p* = 0.23) or the interaction between sex and female longevity (F = 1.31; df1 = 7, df2 = 1160; *p* = 0.19). Moreover, the sex ratios of G2 offspring, representing the ratio of males to females, remained constant across different temperature conditions (F = 1.23; df1 = 7, df2 = 1022; *p* = 0.28), G1 female longevity (F = 1.09; df1 = 1, df2 = 1022; *p* = 0.31), and their interaction (F = 1.51; df1 = 7, df2 = 1022; *p* = 0.17).

### 3.3. Effects of Temperature on G2 Thrips Egg-to-Adult Development Times

Similar to their G1 parent thrips, all G2 *C. fasciatus* offspring individuals successfully completed their development under experimental temperature profiles ranging from 8 to 35 °C (Table 5). Mean egg-to-adult development times of G2 *C. fasciatus* differed with temperature (F = 185.29; df1 = 7, df2 = 2016; *p* < 0.0001; Table 5), but not with sex (F = 2.38; df1 = 1, df2 = 2016; *p* = 0.12; Table 5), and their interactions were insignificant (F = 1.05; df1 = 1, df2 = 2016; *p* = 0.29; Table 5). Egg-to-adult development times of G2 *C. fasciatus* decreased with increasing temperature, irrespective of thrips sex (Table 5).

### 3.4. Fitting Models to Temperature-Driven Development Data

**Figure 1:**
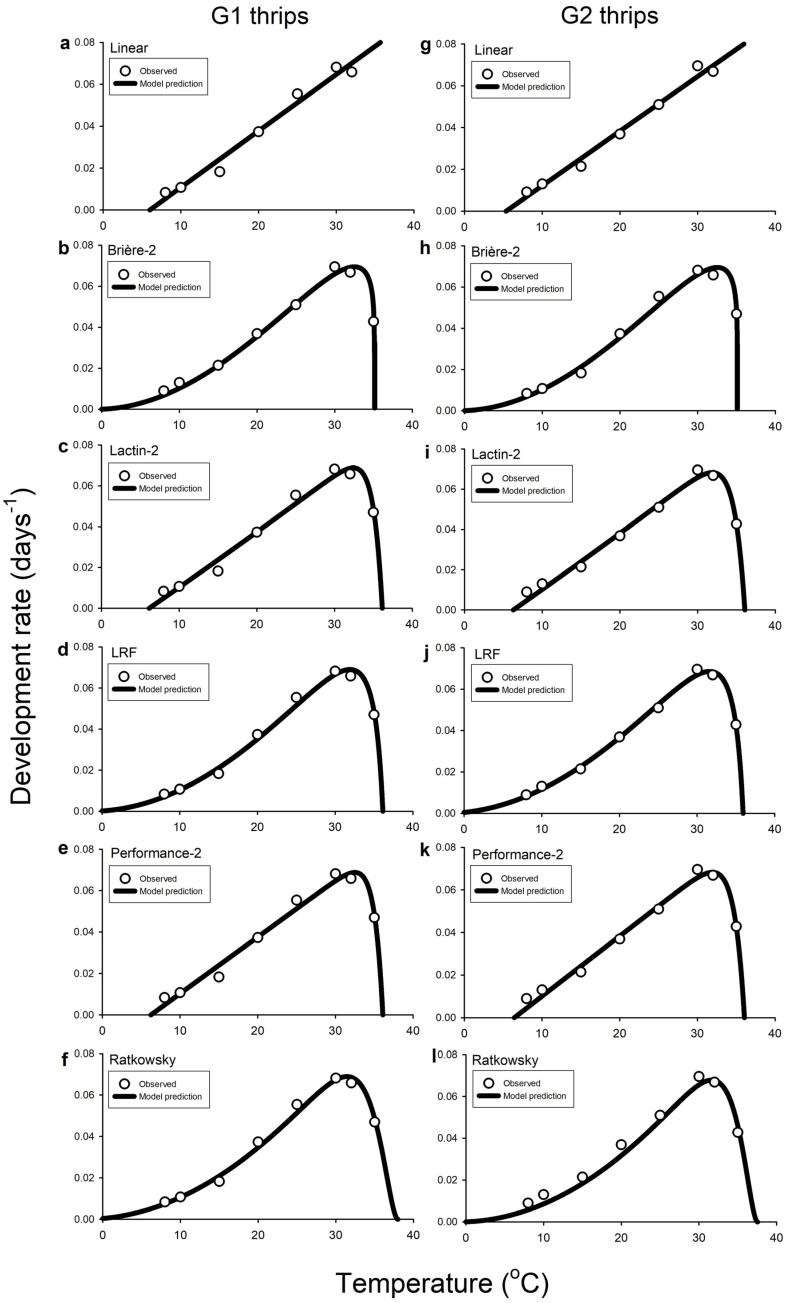
Predicted rate of total development as a function of temperature for generation 1 (G1 (**a**–**f**)) and generation 2 (G2 (**g**–**l**)) of *Caliothrips fasciatus* (pooled males and females) at different eight fluctuating temperature profiles (i.e., 8, 10, 15, 20, 25, 30, 32, or 35 °C) using linear (**a**,**g**), Brière-2 (**b**,**h**), Lactin-2 (**c**,**i**), LRF (**d**,**j**), Performance-2 (**e**,**k**), and Ratkowsky (**f**,**l**) models. In all graphs, the ordinate is the rate of development (1/*D*, in days^−1^), and the abscissa is the temperature (°C). *Open circles* represent the average of recorded data in all graphs. In the linear regression (**a**,**g**), the last data values (i.e., 35 °C) for both the G1 and G2 *Caliothrips fasciatus* development times have been omitted because of deviations from a straight line.

The Ordinary Linear model had *R*^2^_adj_ values of >0.98 for G1 and G2 *C. fasciatus*, indicating very good fit to datasets (Table 6). The regression line predicted lower developmental thresholds (i.e., *T*_min_) of 6.52 °C for G1 thrips (Table 6; Figure 1a) and 5.34 °C for G2 thrips (Table 6; Figure 1g), and the thermal requirements for development completion (i.e., *K*) were 370.37 and 384.61 degree days above these minimum threshold estimates for G1 and G2 *C. fasciatus*, respectively (Table 6; Figure 1a,g). All five nonlinear models exhibited good fits to observed data for G1 and G2 *C. fasciatus*, producing *RSS* values < 0.0001 (Table 6). Compared to the Brière-2, LRF, and Ratkowsky nonlinear equations, the Lactin-2 and Performance-2 models yielded higher *RSS* values for development completions of G1 and G2 *C. fasciatus* (Table 6), indicating slightly poorer fits to both datasets. Nevertheless, estimations of *T*_opt_ (i.e., estimated optimal temperature for development) and *T*_max_ (i.e., upper temperature limit for development) for G1 and G2 *C. fasciatus* were similar among models ranging from 31.34 to 32.52 °C (G1) and 31.19 to 31.92 °C (G2) for *T*_opt_, and 35.12 to 37.98 °C (G1) and 35.07 to 37.63 °C (G2) for *T*_max_, (Table 6; Figure 1b–f,h–l). Considerable divergences in model predictions were observed for values of *T*_min_ (i.e., minimum temperature above which development occurs) for G1 and G2 *C. fasciatus*, which ranged from −4.37 to 6.52 °C for G1 and from –3.77 to 6.28 °C for G2 thrips (Table 6; Figure 1b–f,h–l).

## 4. Discussion

The main driver of critical life history functions for insects is temperature, which strongly influences development, survival, reproduction, and movement [49]. In this study, estimates of *C. fasciatus* pre-imaginal development times, adult longevity, and daily and lifetime fecundity were significantly affected by fluctuating temperature cycles that averaged 8, 10, 15, 20, 25, 30, 32, or 35 °C over a 24 h period (no development was observed at 37 °C). Non-linear model fitting indicated that *T*_min_, *T*_opt_, and *T*_max_ may have laid within the following temperature ranges, 6.23–6.52, 31.19–32.52, and 35.07–37.98 °C, respectively. *T*_min_ predictions from linear regression were in the range of 5.35–6.52 °C and were in agreement with estimates provided by Lactin-2, Brière-2, and Performance-2. Notable outliers with regard to *T*_min_ estimates were negative values (i.e., <0 °C) returned by the Lobry-Rosso-Flandrois (LRF) and Ratkowsky models. Linear regression estimates predicted that 370.37–384.61 degree days above *T*_min_ were required for *C. fasciatus* to complete egg-to-adult development.

With respect to *Caliothrips* spp., temperature affects population phenology [50,51], and, for *C. fasciatus* specifically, overwintering biology, host plant use, pestiferousness, and geographic distribution are influenced strongly by temperature [4,5,6,7]. In addition to *C. fasciatus*, at least two additional pest species of *Caliothrips* are recognized: *C. indicus* (Bagnall), a pest of peanut (*Arachis hypogaea* L.) [50], and *C. phaseoli* (Hood), a pest of bean (*P. vulgaris*) [51]. The effects of varying temperatures on the developmental rates and life history parameters for these two pest species, or other species of *Caliothrips* (excluding *C. fasciatus*), have not been investigated.

Bailey [4] made the first attempt to investigate the relationship between *C. fasciatus* preimaginal development rates, longevity, reproductive biology, and behavior, with constant temperature and humidity. Bailey’s [4] developmental time data for larvae, and propupae and pupae combined, at constants of 15.56, 21.11, 26.67, 32.22, and 37.78 °C, decreased consistently over these five temperatures and followed a linear pattern. These data [4] were not subjected to any statistical or model fitting analyses. As part of this study, analysis of Bailey’s [4] data for these five temperatures was performed using linear regression. Analyses provided estimates of *T*_min_ = 12.15 °C (cf. 5.35–6.52 °C from this study) and a degree-day accumulation of 208.33 (cf. 196.08 from this study) (see Supplementary Materials, Table S1).

This study has expanded considerably on Bailey’s [4] original temperature studies by assessing the effects of nine fluctuating temperatures that averaged 8, 10, 15, 20, 25, 30, 32, 35, and 37 °C (no development was observed at 37 °C) over a 24 h period on development and survivorship rates, fecundities, and sex ratios of *C. fasciatus*. Work presented here has provided estimates of *T*_min_ (i.e., 5.35–6.52 °C), *T*_opt_ (i.e., 31.19–32.52 °C), and *T*_max_ (i.e., 35.07–37.98 °C), which provided important baseline data for assessing effects of accumulative cold stress on *C. fasciatus* fitness. The non-linear relationship between temperature and insect development rates at lower and upper temperature extremes that fluctuate could affect life history traits, and expected outcomes could diverge from those predicted from constant temperature studies [52]. For example, fluctuating temperatures that remained within tolerable temperature ranges could improve fitness, or, alternatively, negative impacts could result from stress accumulations of compounds during successive exposures to stress-inducing temperatures at the lower and upper extremes of the temperature profiles under investigation (i.e., excessive cold or heat exposure) [52]. Therefore, understanding the effects of fluctuating temperatures on *C. fasciatus* is necessary for predictions of insect performances in the field, and, arguably, perhaps most importantly, during prolonged periods of fluctuating low temperatures over winter, when thrips are hibernating inside navel oranges.

One putative explanation for the failure of *C. fasciatus* to establish outside of its native range is the accumulative cold stress hypothesis, which postulates that the fitness of adult thrips is reduced due to the negative aggregative effects of low temperature while overwintering in the navels of oranges in orchards prior to harvest. This stress is further amplified by low refrigerated temperatures experienced during shipping to export destinations [2]. With respect to *C. fasciatus*, *T*_min_ predictions from linear and non-linear analyses completed in this study ranged from ~5 to 7 °C (*T*_min_ estimates from non-linear models ranging from −4.37 to −1.48 °C likely had no biological accuracy (Table 6)) and could be a potential starting temperature range for manipulative field and laboratory experiments assessing the impacts of varying durations of accumulative cold stress on the fitness of adult thrips and the subsequent effects on invasion potential.

In regard to invasive insect pests, the likelihood of invasion and establishment in new areas is affected by the receiving environment, which needs to have a favorable year-round climate and habitat to sustain permanent populations [53]. Meta-analyses indicate that invasive insect species, when compared to non-invasive congeners from similar source-areas, differ significantly with respect to one key thermal requirement, the lower developmental threshold (*T*_min_), which tends to be higher for invasive species when compared to congeneric species not recorded outside of their native range [54]. Additionally, for some species of thrips, there is a negative relationship between *T*_min_ and degree days required for development, such that degree days needed to complete development decline as *T*_min_ increases [55]. These findings suggest that low temperatures may, in part, prevent some insect species, like pest thrips, from successfully invading new areas, and species from warmer areas closer to the tropics with higher *T*_min_ values and lower degree day requirements to complete development may have greater establishment and invasion potentials following introduction into suitable new areas [54,55]. The potential application of these findings [54,55] for assessing the invasion threat posed by *C. fasciatus* warrants investigation.

The genus *Caliothrips* is represented by 23 extant species, of which one, *C. insularis*, originally described from the tropical Caribbean Islands of St. Croix and Cuba and recorded from Venezuela and Brazil and throughout the Caribbean to Florida [56], has been recorded outside of this presumptive native range in Mauritius in the Indian Ocean [56] and in China [57]. Horticultural trade in live plants is the suspected conduit for moving *C. insularis* into new areas [57]. *Caliothrips insularis* is polyphagous, having been collected on various species of grasses, *Cymbogon* sp., *Cyperus esculentus* L., *Lilium* sp., *Saccharum officinarum* L., *Setaria barbata* (Lam.), and *Zea mays* L. [53,55]. *Caliothrips fasciatus* is similarly polyphagous and has been intercepted numerous times over long periods of time (i.e., decades) in shipments of contaminated citrus originating from California in countries (e.g., New Zealand and Australia) at times of the year where the receiving environment would be considered hospitable for surviving thrips [7]. Agricultural exports, like fresh fruit, are recognized as important conduits for movement of invasive insect pests into new areas [58,59]. Yet, despite high levels of propagule pressure over time, *C. fasciatus*, unlike *C. insularis*, has failed to establish outside of its presumptive native range. In this instance, determination of *T*_min_ and degree day requirements for *C. insularis* would be useful for comparison to *C. fasciatus* to ascertain if the invasion potential of *C. fasciatus* would be considered “low” (i.e., lower *T*_min_ and higher degree day accumulation for development) when compared to *C. insularis*.

Another potential factor affecting the invasion potential of *C. fasciatus* is its unusual reproductive biology and the probable existence of cryptic species, one of which is infected with *Wolbachia*, an endosymbiont associated with reproductive incompatibility [8]. Populations of *C. fasciatus* from different geographic source areas are less likely to interbreed and exhibit high levels of pre- and post-mating isolation and outbreeding depression [8]. This situation could exist if imported fruits from different geographic location areas are contaminated with *C. fasciatus* from populations that are reproductively incompatible, and these fruits are commingled prior to packing and shipping. Additionally, *C. fasciatus* is arrhenotokous, and unfertilized eggs produce male offspring. Females need to mate continuously to produce daughters from fertilized eggs [4]. In the absence of males, mated females deplete sperm reserves and produce only male offspring. Sperm depletion may occur within 10 days of mating and subsequent oviposition of fertilized eggs [4]. Unmated female *C. fasciatus*, when isolated individually, fail to produce male offspring, but, when present in groups of five, male offspring are produced by the majority (i.e., 90%) of female groupings [8]. The necessity for a quorum of unmated females to be present together to stimulate oviposition of unfertilized eggs for production of male offspring, which may be able to mate with the foundress population, is an Allee effect and, potentially, a significant barrier to establishment.

## 5. Conclusions

In conclusion, this study is the first to assess the effects of fluctuating temperatures on the development and reproductive biology of *C. fasciatus*, a regular contaminant of fresh citrus fruit exported from California. This thrips has failed to establish outside of its native range, and there are at least two possibilities exist to explain this observation: the accumulation of cold stress and reproductive barriers, neither of which are mutually exclusive, and both of which could affect the fitness of adults that survive the harvesting and export processes. Deeper insight into lack of invasiveness of *C. fasciatus* is possible through additional manipulative studies investigating the effects of stress from low temperature exposures. The accumulative cold stress hypothesis is amenable to laboratory-based experimentation, and the results could be verified by conducting fitness evaluation studies with live adult male and female *C. fasciatus* detected during quarantine inspections of fresh fruit imports at ports of entry.

## Figures and Tables

**Table 1:** Fluctuating temperature profiles utilized for rearing *Caliothrips fasciatus*.

Hour	Mean Temperature (°C)									Photoperiod
	8	10	15	20	25	30	32	35	37	
0100	4	7	11	14	20	25	26	33	30	dark
0200	3	7	10	13	19	24	25	33	30	
0300	3	6	10	13	18	24	25	32	29	
0400	3	6	9	12	17	22	24	32	30	
0500	2	6	9	11	17	21	24	31	27	
0600	2	6	8	11	16	22	24	30	30	light
0700	1	6	8	13	18	24	26	32	34	
0800	2	6	12	17	21	26	29	34	36	
0900	5	8	15	19	23	28	31	36	37	
1000	8	10	17	21	25	30	33	37	40	
1100	11	12	18	22	27	32	35	39	42	
1200	13	13	20	24	29	34	37	40	43	
1300	14	14	21	26	31	35	38	40	44	
1400	15	15	22	27	33	36	40	41	45	
1500	16	15	23	28	33	37	40	41	45	
1600	16	15	23	29	34	38	40	40	45	
1700	15	14	23	29	34	38	40	40	43	
1800	13	13	21	28	33	37	39	38	42	
1900	11	11	18	25	30	36	37	36	40	
2000	9	10	16	23	27	33	34	33	37	dark
2100	8	10	14	21	26	31	32	32	35	
2200	7	9	12	19	24	29	31	31	35	
2300	6	9	11	18	23	27	30	30	35	
2400	6	8	11	16	21	26	28	29	34	
Total Steps	19	14	17	21	21	22	18	19	19	

**Table 2:** Results of generalized linear mixed models examining the effects of sexes, temperatures (i.e., fluctuating temperature regimens that averaged 8, 10, 15, 20, 25, 30, 32, or 35 °C over a 24 h period), and their interactions on development times of first-generation (i.e., G1) *Caliothrips fasciatus* eggs (A), first and second instar larvae (B, C), propupae (D), pupae (E), egg-to-adult (F), and adults (G), reared under fluctuating temperature regimes.

(A) Eggs (Development)	Num df	Den df	F	*p*
Sex (S)	1	168	0.84	0.3612
Temperature (T)	7	168	187.21	<0.0001 *
S × T	7	168	0.31	0.9541
(B) 1st Instar Larvae (Development)	Num df	Den df	F	*p*
Sex (S)	1	168	0.17	0.6795
Temperature (T)	7	168	2.54	0.0164 *
S × T	7	168	0.08	0.9992
(C) 2nd Instar Larvae (Development)	Num df	Den df	F	*p*
Sex (S)	1	168	0.01	0.9343
Temperature (T)	7	168	102.96	<0.0001 *
S × T	7	168	0.16	0.9922
(D) Propupae (Development)	Num df	Den df	F	*p*
Sex (S)	1	168	0.01	0.9152
Temperature (T)	7	168	59.36	<0.0001 *
S × T	7	168	0.48	0.8511
(E) Pupae (Development)	Num df	Den df	F	*p*
Gender (S)	1	168	0.01	0.9251
Temperature (T)	7	168	106.53	<0.0001 *
S × T	7	168	0.29	0.9557
(F) Egg-to-Adult (Development)	Num df	Den df	F	*p*
Sex (G)	1	168	0.31	0.5831
Temperature (T)	7	168	408.51	<0.0001 *
S × T	7	168	0.35	0.9288
(G) Adult (Longevity)	Num df	Den df	F	*p*
Sex (S)	1	168	0.04	0.8501
Temperature (T)	7	168	14.16	<0.0001 *
S × T	7	168	0.24	0.9746

* Indicates significance at the 0.05 level.

**Table 3:** Mean development times (mean days ± SE) of first-generation (G1) *Caliothrips fasciatus* eggs, first and second instar larvae, propupae, pupae, combined egg-to-adult, and adult longevity reared under eight fluctuating temperature regimes that averaged 8, 10, 15, 20, 25, 30, 32, or 35 °C, over a 24 h period.

Temp. (°C)	Mean Development Times (Mean Days ± SE)													
	Eggs		1st Instar Larvae		2nd Instar Larvae		Propupae		Pupae		Egg-to-Adult		Adults	
	Female	Male	Female	Male	Female	Male	Female	Male	Female	Male	Female	Male	Female	Male
8	55.33 ± 9.68 ^a^ (3)	47.33 ± 10.34 ^a^ (3)	2.67 ± 0.33 ^a^ (3)	2.33 ± 0.33 ^a^ (3)	30.33 ± 2.73 ^a^ (3)	28.67 ± 2.69 ^a^ (3)	9.33 ± 2.85 ^a^ (3)	13.67 ± 3.53 ^a^ (3)	28.33 ± 2.61 ^a^ (3)	29.67 ± 2.67 ^a^ (3)	125.33 ± 12.66 ^a^ (3)	120.67 ± 14.31 ^a^ (3)	2.67 ± 1.67 ^d^ (3)	1.67 ± 0.67 ^d^ (3)
10	52.57 ± 1.58 ^a^ (7)	48.25 ± 1.44 ^a^ (4)	2.28 ± 0.36 ^a^ (7)	2.21 ± 0.18 ^a^ (4)	21.29 ± 2.43 ^b^ (7)	19.5 ± 3.95 ^b^ (4)	5.43 ± 0.57 ^b^ (7)	4.25 ± 0.85 ^b^ (4)	10.57 ± 1.79 ^b^ (7)	8.01 ± 1.58 ^b^ (4)	92.14 ± 7.17 ^b^ (7)	82.75 ± 10.41 ^b^ (4)	28.14 ± 8.57 ^a^ (7)	25.75 ± 5.98 ^a^ (4)
15	23.92 ± 0.29 ^b^ (11)	23.62 ± 0.67 ^b^ (10)	2.25 ± 0.29 ^a^ (11)	2.29 ± 0.27 ^a^ (10)	7.42 ± 0.49 ^c^ (11)	7.41 ± 0.87 ^c^ (10)	11.25 ± 0.85 ^a^ (11)	11.21 ± 1.12 ^a^ (10)	9.23 ± 0.79 ^b^ (11)	9.39 ± 0.93 ^b^ (10)	53.17 ± 1.31 ^c^ (11)	53.21 ± 2.11 ^c^ (10)	23.58 ± 3.96 ^a^ (11)	20.81 ± 3.71 ^a^ (10)
20	16.23 ± 0.28 ^c^ (22)	15.72 ± 0.31 ^c^ (14)	1.36 ± 0.12 ^b^ (22)	1.37 ± 0.11 ^b^ (14)	4.65 ± 0.28 ^d^ (22)	4.72 ± 0.41 ^d^ (14)	1.63 ± 0.15 ^c^ (22)	1.71 ± 0.17 ^c^ (14)	3.68 ± 0.24 ^c^ (22)	3.79 ± 0.35 ^c^ (14)	27.54 ± 0.39 ^d^ (22)	27.28 ± 0.51 ^d^ (14)	13.18 ± 3.67 ^b^ (22)	12.57 ± 4.04 ^b^ (14)
25	11.13 ± 0.46 ^d^ (20)	11.35 ± 0.85 ^d^ (16)	1.33 ± 0.18 ^b^ (20)	1.32 ± 0.42 ^b^ (16)	3.89 ± 0.28 ^d^ (20)	3.59 ± 0.24 ^d^ (16)	1.45 ± 0.12 ^c^ (20)	1.23 ± 0.1 ^c^ (16)	2.09 ± 0.23 ^de^ (20)	2.32 ± 0.33 ^de^ (16)	20.19 ± 0.52 ^e^ (20)	20.23 ± 0.79 ^e^ (16)	9.12 ± 2.97 ^b^ (20)	9.35 ± 1.96 ^b^ (16)
30	7.09 ± 0.36 ^e^ (12)	6.98 ± 0.45 ^e^ (11)	1.28 ± 0.14 ^b^ (12)	1.29 ± 0.15 ^b^ (11)	2.36 ± 0.24 ^e^ (12)	2.29 ± 0.21 ^e^ (11)	1.25 ± 0.15 ^c^ (12)	1.29 ± 0.16 ^c^ (11)	1.25 ± 0.13 ^e^ (12)	1.24 ± 0.14 ^e^ (11)	14.25 ± 0.61 ^f^ (12)	14.46 ± 0.83 ^f^ (11)	5.08 ± 0.75 ^c^ (12)	4.91 ± 0.86 ^c^ (11)
32	7.82 ± 0.69 ^e^ (11)	8.11 ± 0.75 ^e^ (10)	1.17 ± 0.17 ^b^ (11)	1.21 ± 0.23 ^b^ (10)	2.09 ± 0.31 ^e^ (11)	2.11 ± 0.45 ^e^ (10)	1.21 ± 0.21 ^c^ (11)	1.21 ± 0.22 ^c^ (10)	1.75 ± 0.27 ^e^ (11)	1.76 ± 0.35 ^e^ (10)	14.73 ± 0.72 ^f^ (11)	15.01 ± 0.92 ^f^ (10)	3.73 ± 1.05 ^cd^ (11)	3.82 ±1.73 ^cd^ (10)
35	10.71 ± 0.58 ^d^ (20)	10.03 ± 0.91 ^d^ (10)	1.38 ± 0.23 ^b^ (20)	1.31 ± 0.27 ^b^ (10)	3.08 ± 0.32 ^d^ (20)	3.32 ± 0.49 ^d^ (10)	3.45 ± 0.39 ^b^ (20)	3.4 ± 0.45 ^b^ (10)	2.92 ± 0.32 ^cd^ (20)	3.16 ± 0.57 ^cd^ (10)	20.55 ± 0.39 ^e^ (20)	20.51 ± 0.82 ^e^ (10)	2.51 ± 0.41 ^d^ (20)	2.23 ± 0.49 ^d^ (10)
*p*	<0.0001		0.0164		<0.0001		<0.0001		<0.0001		<0.0001		<0.0001	

Means within the same column followed by the same letter were not significantly different at α = 0.05 (LSMEANS), and number in round brackets (i.e., ( )) denotes number of individuals.

**Table 4:** Mean estimated lifetime fecundity, and numbers of emerged second-generation (G2) *C. fasciatus* males and females (±SE), produced by first generation (G1) female *Caliothrips fasciatus* reared under eight fluctuating temperature profiles that averaged 8, 10, 15, 20, 25, 30, 32, or 35 °C over a 24 h period.

Temperature (°C)	Mean Lifetime Fecundity * of G1 Females and Sex of Progeny		
	Mean No. of G2 Larvae ± SE	Mean No. Adult G2 Thrips ± SE	
		Female	Male
8	1.33 ± 0.88 ^d^ [3] {4}	0.67 ± 0.33 [3] (2)	0.67 ± 0.33 [3] (2)
10	16.75 ± 8.52 ^bc^ [4] {67}	11.75 ± 5.52 [4] (47)	4.51 ± 2.12 [4] (18)
15	24.89 ± 9.57 ^bc^ [11] {273}	12.09 ± 4.57 [11] (122)	12.54 ± 4.03 [11] (138)
20	39.72 ± 11.76 ^ab^ [14] {556}	20.71 ± 3.76 [14] (290)	18.53 ± 6.14 [14] (259)
25	50.82 ± 8.24 ^a^ [11] {559}	27.91 ± 4.24 [11] (307)	22.45 ± 2.57 [11] (247)
30	19.25 ± 5.09 ^bc^ [12] {231}	7.53 ± 3.09 [12] (143)	4.53 ± 1.35 [12] (86)
32	15.73 ± 7.12 ^bc^ [11] {173}	9.68 ± 4.12 [11] (107)	3.27 ± 1.99 [11] (36)
35	13.53 ± 6.14 ^c^ [15] {203}	8.23 ± 2.14 [15] (123)	5.27 ± 1.09 [15] (78)

* Mean lifetime female fecundity was estimated as the average total number of larvae that emerged under that temperature regimen. Means within the same column followed by the same letter were not significantly different α = 0.05 (LSMEANS); number in square brackets (i.e., [ ]) denotes number of G1 female thrips used at each temperature profile. Number in parentheses (i.e., { } denotes total number of eggs produced by G1 female thrips at each temperature profile. Number in round brackets (i.e., ( )) denotes total number of G2 thrips successfully reared to adults at each temperature profile (see Table 5 for more details).

**Table 5:** Mean egg-to-adult development time in days (± SE) for second-generation (G2) *Caliothrips fasciatus* females and males reared under eight fluctuating temperature profiles that averaged 8, 10, 15, 20, 25, 30, 32, or 35 °C over a 24 h period.

	G2 Egg-to-Adult Development Times (Mean Days ± SE)							
	8 °C	10 °C	15 °C	20 °C	25 °C	30 °C	32 °C	35 °C
Males	107.50 ± 8.50 ^a^ (2)	78.44 ± 7.82 ^b^ (18)	47.82 ± 0.76 ^c^ (138)	27.39 ± 0.19 ^d^ (259)	19.81 ± 0.12 ^e^ (247)	14.24 ± 0.35 ^e^ (86)	14.72 ± 0.64 ^e^ (36)	23.28 ± 0.21 ^d^ (78)
Females	110.00 ± 0.50 ^a^ (2)	74.77 ± 7.11 ^b^ (47)	45.34 ± 0.43 ^c^ (122)	26.79 ± 0.19 ^d^ (290)	19.39 ± 0.31 ^e^ (307)	14.51 ± 0.17 ^e^ (143)	15.19 ± 0.23 ^e^ (112)	23.41 ± 0.13 ^d^ (123)

Means followed by the same letter within the same row are not significantly different at α = 0.05; number in round brackets (i.e., ( )) denotes number of individuals used. Standard errors are based upon the pooled variance and the individual sample sizes.

**Table 6:** Mathematical model equations, model parameters, parameter estimates, and goodness-of-fit metrics for six functions describing the relationship between temperature and development rates (*D*_r_) of first-generation (G1) and second-generation (G2) *Caliothrips fasciatus* (male and female data combined) reared under eight fluctuating temperature profiles that averaged 8, 10, 15, 20, 25, 30, 32, or 35 °C over a 24 h period.

Model	Model Equation ^†^	Parameter	Parameter Estimate		References
			G1 Thrips	G2 Thrips	
Ordinary Linear ^‡^	$D_{r}=a+bT$	a	−0.0176	−0.0139	[28]
		b	0.0027	0.0026	
		*K* (degree days) ^‡^	370.37	384.61	
		*T*_min_ ^‡^	6.52	5.35	
		*R* ^2^_adj_	0.9813	0.9849	
Lactin-2 (Logan-Lactin)	$D_{r}=\lambda +e^{\rho T}-e^{\left(\rho T_{u}-(T_{u}-T)/\delta T\right)}$	λ	−1.0161	−1.0179	[33,34]
		*ρ*	0.0026	0.0027	
		*δ*	1.1561	1.3782	
		*T*_min_ *	6.52	6.28	
		*T*_u_ (*T*_max_ *)	36.14	37.63	
		*T*_opt_ *	32.51	31.79	
		*RSS*	0.000016	0.000011	
Brière-2	$D_{r}={aT\left(T-T_{\min}\right)(T_{\max}-T)}^{1/b}$	*a*	0.000054	0.000051	[32]
		*b*	6.4394	5.8481	
		*T*_min_	−1.37	−2.74	
		*T*_max_	35.12	35.07	
		*T*_opt_ *	32.52	31.92	
		*RSS*	0.000008	0.000002	
Lobry-Rosso-Flandrois (LRF)	$D_{r}=\mu_{\mathrm{opt}}\frac{{\left(T-T_{\max})(T-T_{\min}\right)}^{2}}{(T_{\mathrm{opt}}-T_{\min})\left[\left(T_{\mathrm{opt}}-T_{\min}\right)\left({T-T}_{\mathrm{opt}}\right)-(T_{\mathrm{opt}}-T_{\max})(T_{\mathrm{opt}}+T_{\min}-2T)\right]}$	*μ*_opt_	0.0689	0.0685	[35,36]
		*T*_min_	−1.48	−2.49	
		*T*_max_	36.13	35.92	
		*T*_opt_	31.89	31.59	
		*RSS*	0.000006	0.000005	
Performance-2	$D_{r}=b\left(T-T_{\min}\right)(1-e^{c\left(T-T_{\max}\right)})$	*T*_min_	6.52	6.23	[37,38]
		*T*_max_	36.09	36.05	
		*T*_opt_ *	32.39	31.89	
		*b*	0.0027	0.0028	
		*c*	0.8807	0.7188	
		*RSS*	0.000014	0.000012	
Ratkowsky	$\sqrt{D_{r}}=b\left(T-T_{\min}\right)(1-e^{c\left(T-T_{\max}\right)});$ $D_{r}={\left[b\left(T-T_{\min}\right)\left(1-e^{c\left(T-T_{\max}\right)}\right)\right]}^{2}$	*b*	0.0083	0.0081	[39]
		*c*	0.4112	0.4417	
		*T*_min_	−4.37	−3.77	
		*T*_max_	37.98	37.49	
		*T*_opt_ *	31.34	31.19	
		*RSS*	0.000005	0.000003	

^†^ In all models, *T* is the temperature in degrees Celsius (°C), and *D*_r_ is the development rate at temperature *T*. In the Ratkowsky, Lactin-2, and Performance-2 models, *e* denotes the base of the natural logarithms. Other symbols denote model parameters (see corresponding references for full description of models and parameters). ^‡^ Linear regression was used to calculate the theoretical minimum developmental threshold (*T*_min_ = −*a*/*b*, where *a* is the development rate when *T* = 0 °C and *b* is the slope), and thermal constant or degree days necessary for completion of development (*K* = 1/*b*). * In all nonlinear models, except in the LRF model, the optimum development temperature (*T*_opt_) was estimated from the development curve peak (where *D*_r_ = max). In the Lactin-2 and Beta models, the theoretical minimum and maximum developmental thresholds (*T*_min_ and *T*_max_) were measured at the sites of intersection between the curve and temperature axis (where *D*_r_ = 0). Within the LRF model, *μ*_opt_ and *T*_opt_ are parameters inherent to the model; *T*_opt_ represents the optimal temperature for insect growth, whereas *μ*_opt_ corresponds to the growth rate achieved under those ideal conditions.

## Data Availability

Data are available upon request.

## Supplementary Materials

The following supporting information, can be downloaded at: https://www.mdpi.com/article/10.3390/insects14070641/s1. Table S1. Estimated parameters and *R*^2^_adj_ values of the linear model for describing the relationship between development rate (*D*_r_) and temperature for immature stages (i.e., larvae and, propupae and pupae combined) of *Caliothrips fasciatus* (male and female data combined) under constant (i.e., 15.56, 21.11, 26.67, 32.22, and 37.78 °C).
