# Supplementary material for: Effects of Temperature on the Developmental and Reproductive Biology of North American Bean Thrips, Caliothrips fasciatus (Pergande) (Thysanoptera: Thripidae: Panchaetothripinae)
Source: Insects. 2023 Jul 15;14(7):641. doi: 10.3390/insects14070641 (PMC10380338; doi:10.3390/insects14070641)
Supplement: Supplementary file 1 [file insects-14-00641-s001.zip › insects-2484256-supplementary.pdf]

Table S1. Estimated parameters and R^2_{adj} values of the linear model for describing the relationship between development rate (D_r) and temperature for immature stages (larvae and pupae) of *Caliothrips fasciatus* (male and female data combined) under constant (data are from Bailey [1933]) and fluctuating temperature (this study) profiles.

Model	Model Equation	Parameter	Parameter Estimate		Reference
			Fluctuating (This study)	Constant (Bailey 1933)	
Ordinary Linear	$D_r = a + bT$	a	-0.0321	-0.0583	Campbell et al. (1974)
		b	0.0051	0.0048	
		K (degree-days)	196.08	208.33	
		T_{min}	6.29	12.15	
		R^2_{adj}	0.9821	0.9784	

See corresponding reference for full description of model parameters

For both datasets, temperatures at which development rate of *C. fasciatus* larvae and pupae deviated from rectilinearity were omitted from analyses (i.e., development rate at 20 and 37 °C in this study; all temperature dependent data from Bailey's [1933] study aligned with the linear portion of the dataset).
